# Supplementary material for: Remoscope: a label-free imaging cytometer for malaria diagnostics
Source: medRxiv. 2024 Nov 13:2024.11.12.24317184. Preprint. [Version 1] doi: 10.1101/2024.11.12.24317184 (PMC11601755; doi:10.1101/2024.11.12.24317184)
Supplement: 1 [file NIHPP2024.11.12.24317184V1-supplement-1.pdf]

medRxiv preprint doi: https://doi.org/10.1101/2024.11.12.24317184; this version posted November 13, 2024. The copyright holder for this preprint (which was not certified by peer review) is the author/funder, who has granted medRxiv a license to display the preprint in perpetuity.
It is made available under a CC-BY 4.0 International license .

Supplementary Information

Supplementary Videos

Supplementary videos can be downloaded here:
Supplementary_vids

Supplementary Video 1 | Overview of the Remoscope workflow. A video demonstrating the workflow from fingertip blood draw, sample loading, metadata entry, cartridge loading, experiment progress, and summary report.

Supplementary Videos 2-17 | Titration data, points 1-16. The first 30 seconds of each titration dataset from Figure 3 are shown. The first titration point (highest parasitemia, Supplementary Video 2) was diluted twofold in diluent buffer in order to aid with flowing high concentrations of late-stage parasites.

Supplementary Video 18 | High clinical parasitemia. Blood from a participant from the Ugandan cohort with a parasitemia of 654,045 parasites/μL by qPCR. A white blood cell containing phagocytosed hemozoin fragments is visible crossing the field of view.

Supplementary Video 19 | Sickle Cell Disease. Blood from a participant from the Ugandan cohort with sickle cell disease is shown. Various RBC morphologies are present including sickled cells, poikilocytes, target cells, among other non-discocytes.

Supplementary Video 20 | Agglutination. A video showing *Pf* parasites cultured in red blood cells from one donor, diluted into healthy whole blood from a second, non typed-matched donor. Plasma from the second donor is seen to agglutinate RBCs from the first donor, forming large cross-linked rafts of cells. In conditions such as cold agglutination (31), a similar effect is caused by autoantibodies.

Supplementary Video 21 | Abnormal RBCs in a Ugandan cohort participant. Target cells, echinocytes, stomatocytes, and other abnormal RBC morphologies in a participant with normal genotype (not SCD), who also exhibited a parasitemia of 6730.4 parasites/μL (by aPCR).

medRxiv preprint doi: https://doi.org/10.1101/2024.11.12.24317184; this version posted November 13, 2024. The copyright holder for this preprint (which was not certified by peer review) is the author/funder, who has granted medRxiv a license to display the preprint in perpetuity.
It is made available under a CC-BY 4.0 International license .

Supplementary Figures

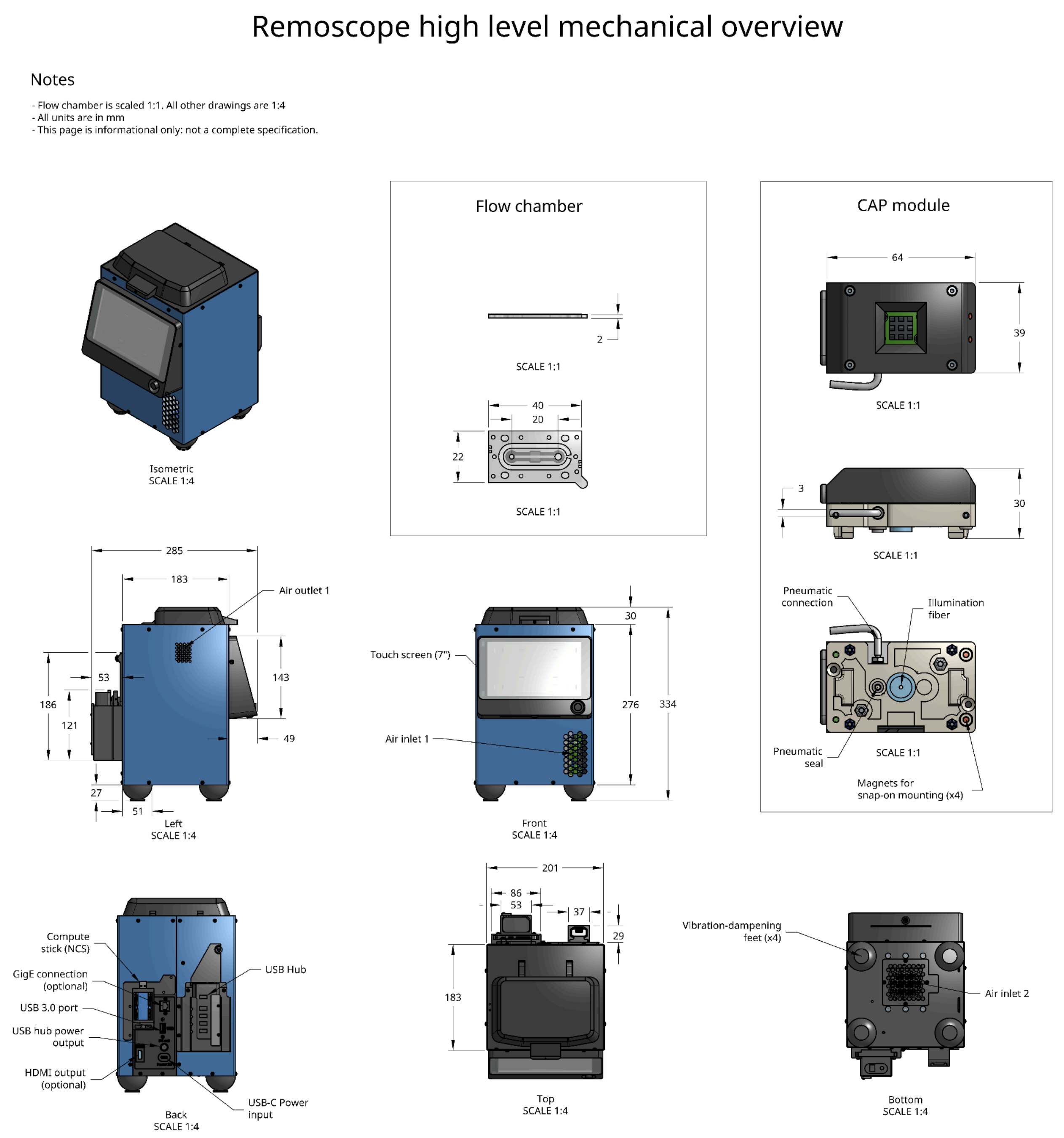

Supplementary Fig. 1 | Overall mechanical design of the system, user-facing elements only. High-level overview of the system including overall dimensions of key components including the instrument exterior dimensions, the CAP module, and the flow cartridge.

medRxiv preprint doi: https://doi.org/10.1101/2024.11.12.24317184; this version posted November 13, 2024. The copyright holder for this preprint (which was not certified by peer review) is the author/funder, who has granted medRxiv a license to display the preprint in perpetuity.
It is made available under a CC-BY 4.0 International license .

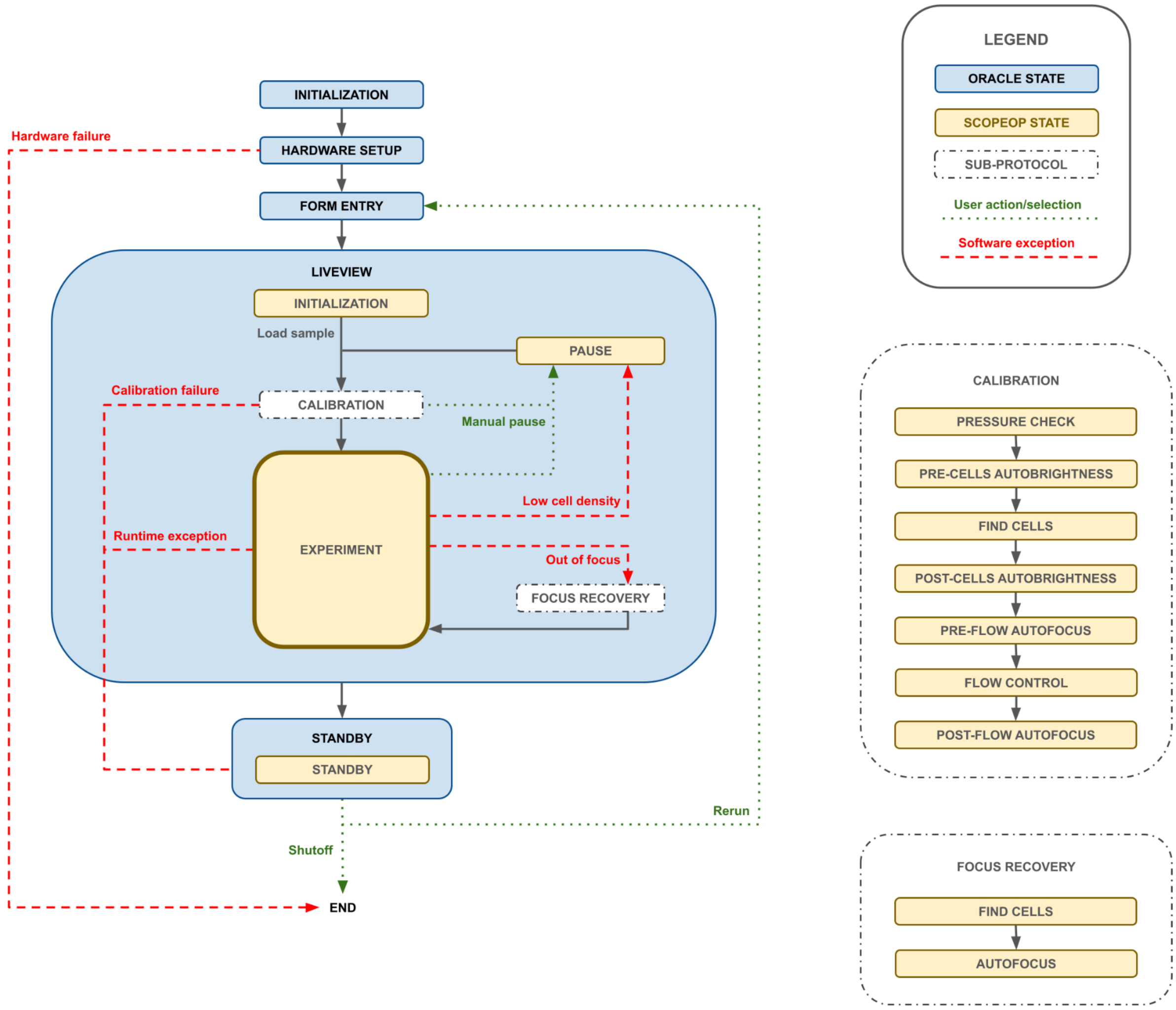

Supplementary Fig. 2 | Remoscope software state machine flowchart. Two state machines were used to coordinate the sequence of events in an experiment. “Oracle” is a state machine class used to control the high level flow from standby, setup, form entry, run sample, and run completion states. “ScopeOp” (owned by Oracle), was used to control real-time dynamics of the hardware. All hardware I/O was managed by ScopeOp. In the diagram, Oracle states are denoted by blue boxes with bold text labels, and ScopeOp states are denoted by yellow boxes with regular text labels. Software-triggered state transitions are indicated by red dotted arrows, and user-triggered transitions are indicated by black solid arrows.

medRxiv preprint doi: https://doi.org/10.1101/2024.11.12.24317184; this version posted November 13, 2024. The copyright holder for this preprint (which was not certified by peer review) is the author/funder, who has granted medRxiv a license to display the preprint in perpetuity.
It is made available under a CC-BY 4.0 International license .

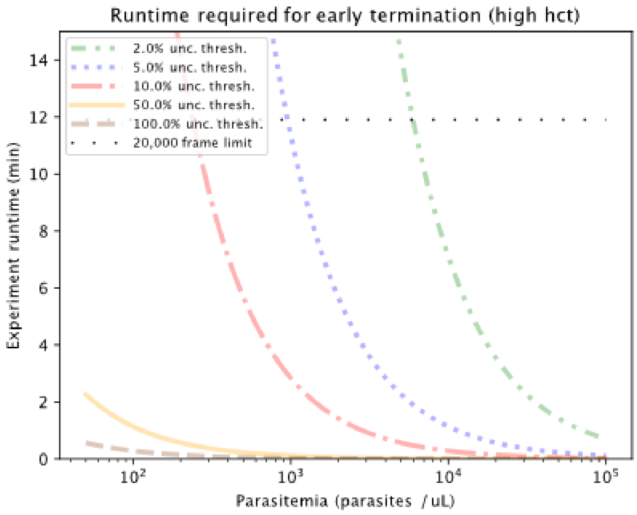

Supplementary Fig. 3 | Remoscope early termination estimates for the undiluted blood assay (high hematocrit, (hct)). Based on Poisson counting statistics only, the experiment run duration was computed using the average rate of cellular throughput for various target relative uncertainty thresholds, as a function of parasitemia. These estimates do not include sources of correlated error.

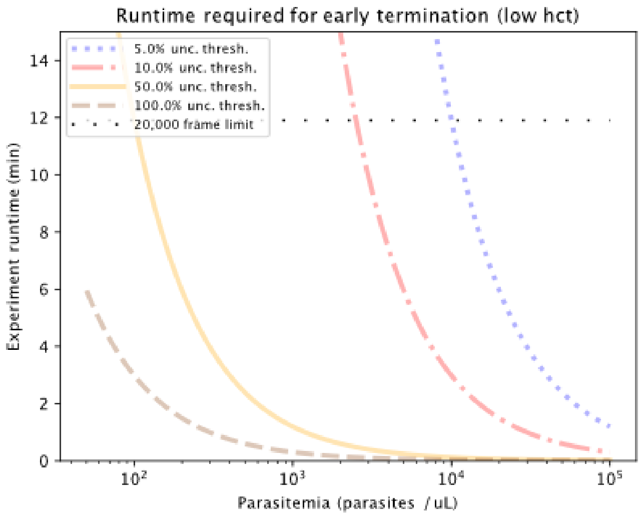

Supplementary Fig. 4 | Remoscope early termination estimates for the diluted blood assay (low hct). Based on Poisson counting statistics only, the experiment run duration was computed using the average rate of cellular throughput for various target relative uncertainty thresholds, as a function of parasitemia. These estimates do not include sources of correlated error.

medRxiv preprint doi: https://doi.org/10.1101/2024.11.12.24317184; this version posted November 13, 2024. The copyright holder for this preprint (which was not certified by peer review) is the author/funder, who has granted medRxiv a license to display the preprint in perpetuity.
It is made available under a CC-BY 4.0 International license .

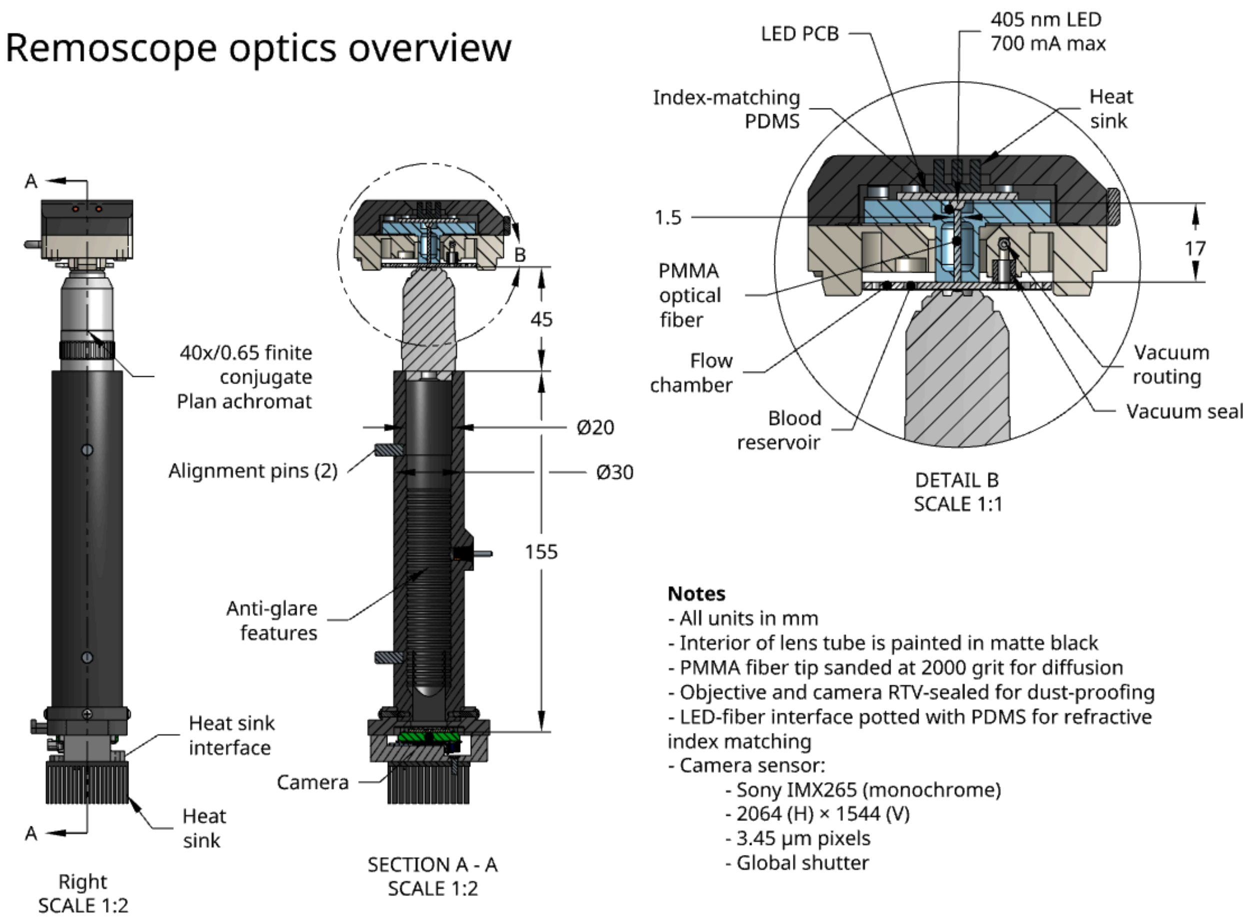

Supplementary Fig. 5 | Remoscope optics. Remoscope optics consist of a simple finite conjugate brightfield microscope with 405 nm excitation, provided by a near UV LED that is coupled into a PMMA optical fiber. Polydimethylsiloxane (PDMS) was used as a potting agent to improve coupling into the fiber, providing a 30% increase in efficiency. The Condenser And Pneumatic module (CAP) houses the excitation as well as the vacuum interface to the flow cartridge. Imaging is performed using a Plan Achromat 40× objective with a numerical aperture of 0.65. Images were acquired using an AVT Alvium 1800 U-319 machine vision camera with a monochrome global shutter Sony IMX265 sensor. A silicone RTV compound was used to seal all objective and camera thread interfaces in order to prevent entry of dust into the optics.

medRxiv preprint doi: https://doi.org/10.1101/2024.11.12.24317184; this version posted November 13, 2024. The copyright holder for this preprint (which was not certified by peer review) is the author/funder, who has granted medRxiv a license to display the preprint in perpetuity.
It is made available under a CC-BY 4.0 International license .

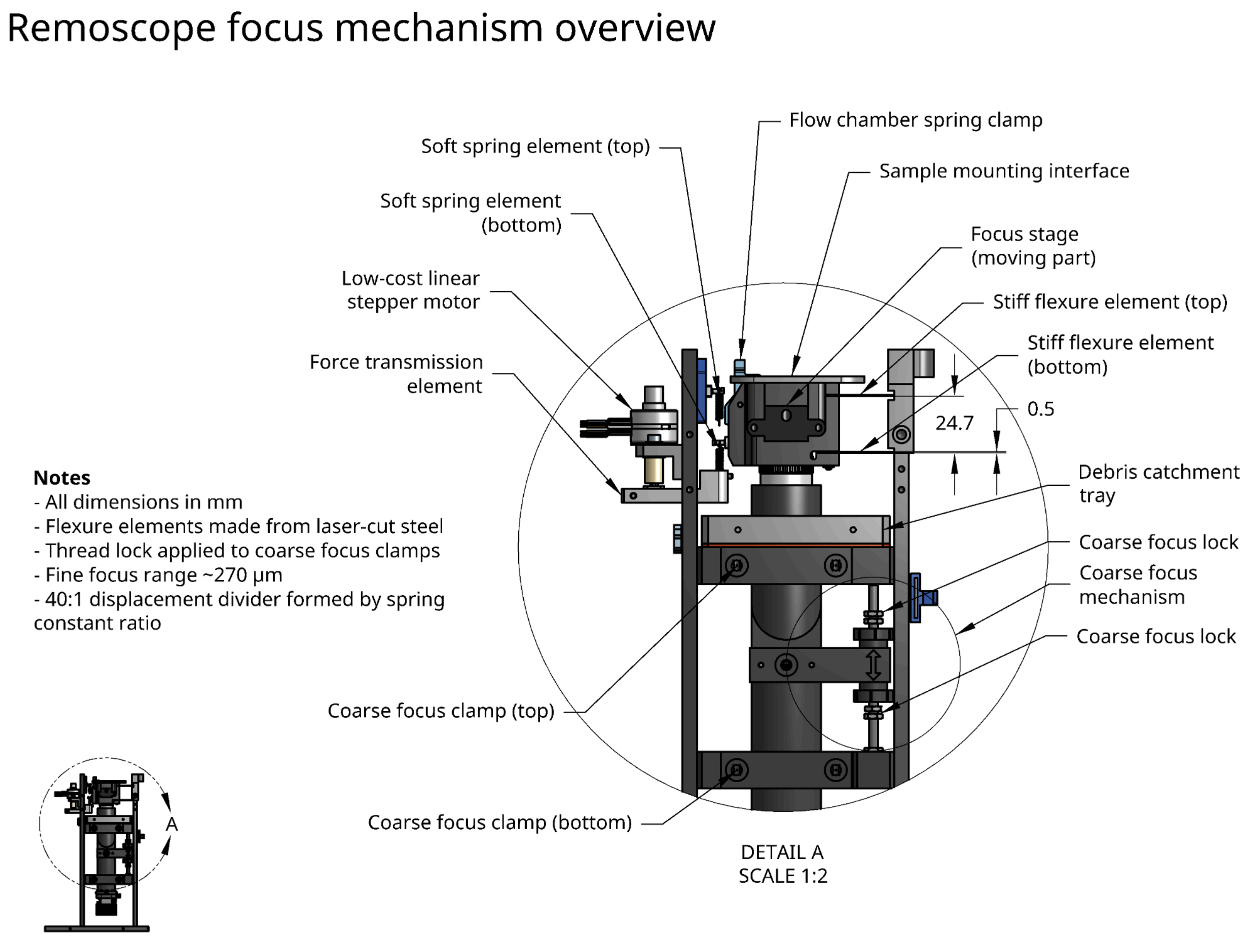

Supplementary Fig. 6 | Design of the focus stage. Remoscope has both coarse and fine focus mechanisms. The coarse mechanism moves the optics sub-assembly by rotating translating thumbscrews vertically along a threaded rod, setting and locking the sliding range of motion. Adjustments on the order of ~5-10 microns can be made via careful rotation of the lower thumb screw, allowing coarse finding of the sample focus during assembly of the instrument (not for routine use). The fine focus mechanism consists of a 40:1 "displacement divider" analogous to a voltage divider: the ratio of spring constants (soft/stiff elements) defines a relative motion amplitude of the sample stage with respect to the linear stepper motor. The total displacement range is ~270 µm with a step size of ~0.3 µm.

medRxiv preprint doi: https://doi.org/10.1101/2024.11.12.24317184; this version posted November 13, 2024. The copyright holder for this preprint (which was not certified by peer review) is the author/funder, who has granted medRxiv a license to display the preprint in perpetuity.
It is made available under a CC-BY 4.0 International license .

Remoscope pneumatic module overview

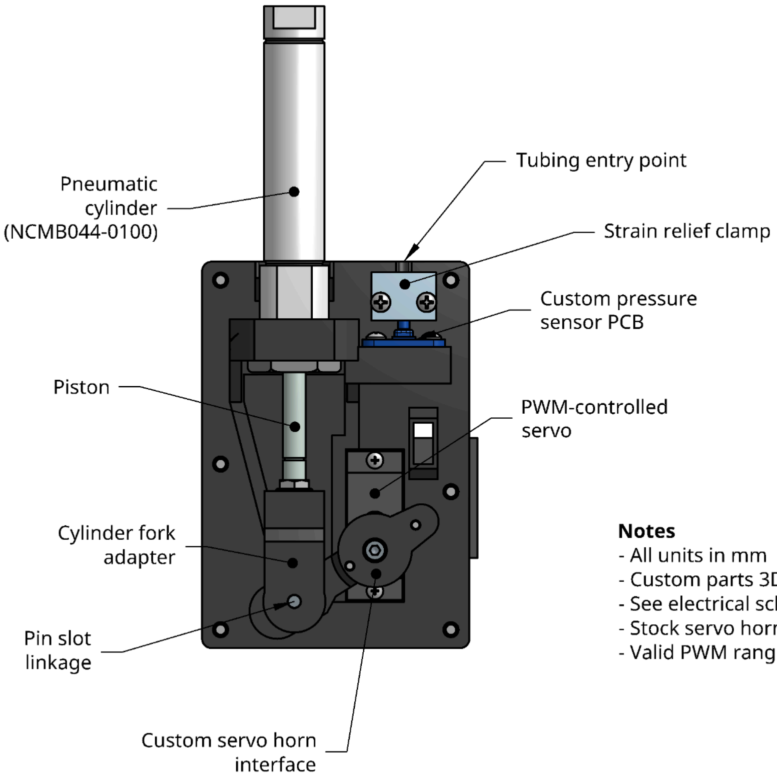

Notes
- All units in mm
- Custom parts 3D printed in tough PLA
- See electrical schematics for custom PCB design
- Stock servo horn cut to fit inside custom interface
- Valid PWM range calibrated for each unit

Supplementary Fig. 7 | Design of the pneumatic module. The pneumatic module uses a PWM-controlled servo motor to adjust the piston of a commercial pneumatic cylinder, via a pin slot linkage formed by a 3D-printed interface to the servo horn and a pin mounted to the cylinder fork adapter. Polyethylene tubing is connected to the top of the cylinder, with a tee junction to split off the connection to the pressure sensor. The fluted, absolute pressure sensor is mounted on a custom PCB. Each module has a valid PWM range that is calibrated on-scope using an automated script in the ulc_mm_scope software package.

medRxiv preprint doi: https://doi.org/10.1101/2024.11.12.24317184; this version posted November 13, 2024. The copyright holder for this preprint (which was not certified by peer review) is the author/funder, who has granted medRxiv a license to display the preprint in perpetuity.
It is made available under a CC-BY 4.0 International license .

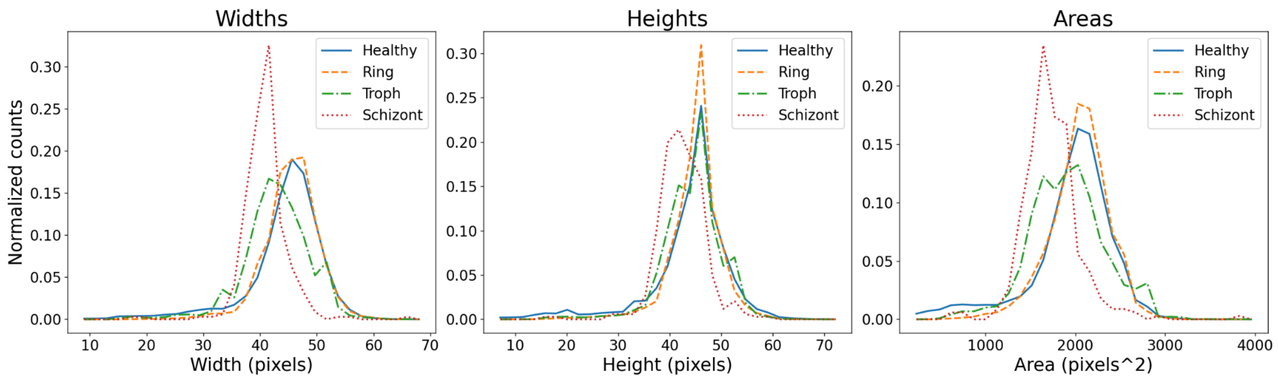

Supplementary Fig. 8 | Cell size histograms. Histograms of bounding box dimensions of fully-annotated cells by class type from the training data, containing both cultured and clinical samples. Each histogram is normalized to the total count for its cell type.

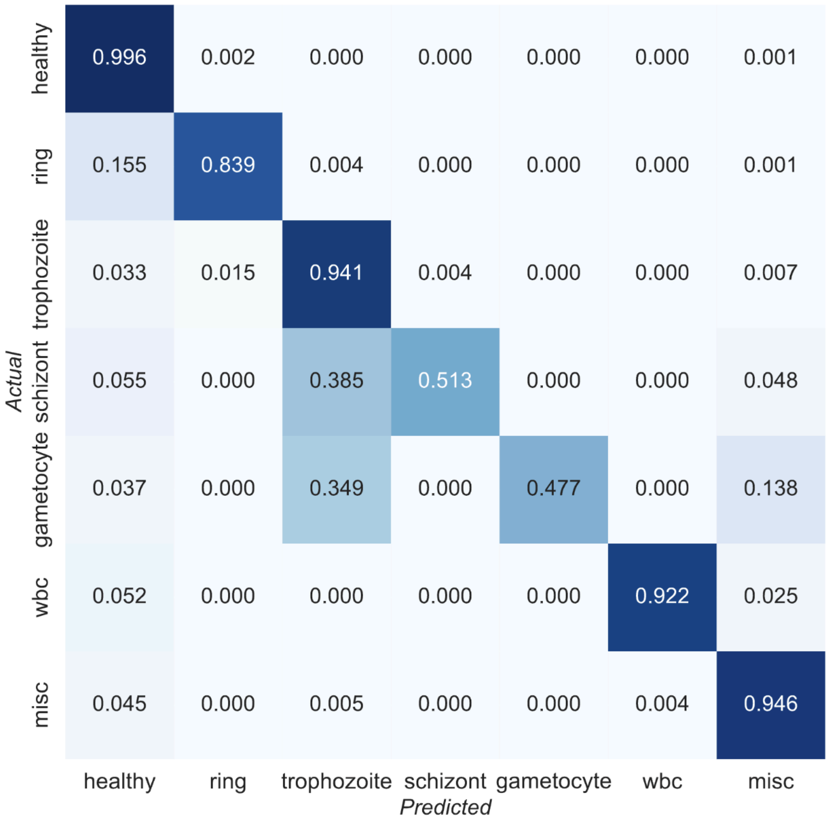

Actual \ Predicted	healthy	ring	trophozoite	schizont	gametocyte	wbc	misc
healthy	0.996	0.002	0.000	0.000	0.000	0.000	0.001
ring	0.155	0.839	0.004	0.000	0.000	0.000	0.001
trophozoite	0.033	0.015	0.941	0.004	0.000	0.000	0.007
schizont	0.055	0.000	0.385	0.513	0.000	0.000	0.048
gametocyte	0.037	0.000	0.349	0.000	0.477	0.000	0.138
wbc	0.052	0.000	0.000	0.000	0.000	0.922	0.025
misc	0.045	0.000	0.005	0.000	0.000	0.004	0.946

Supplementary Fig. 9 | YOGO confusion matrix. The row-normalized confusion matrix was generated by comparing YOGO predictions to human-annotated Remoscope images on a test partition of the data (not seen during training). 'Actual' denotes human-annotated labels and 'Predicted' denotes YOGO model predictions.

medRxiv preprint doi: https://doi.org/10.1101/2024.11.12.24317184; this version posted November 13, 2024. The copyright holder for this preprint (which was not certified by peer review) is the author/funder, who has granted medRxiv a license to display the preprint in perpetuity.
It is made available under a CC-BY 4.0 International license .

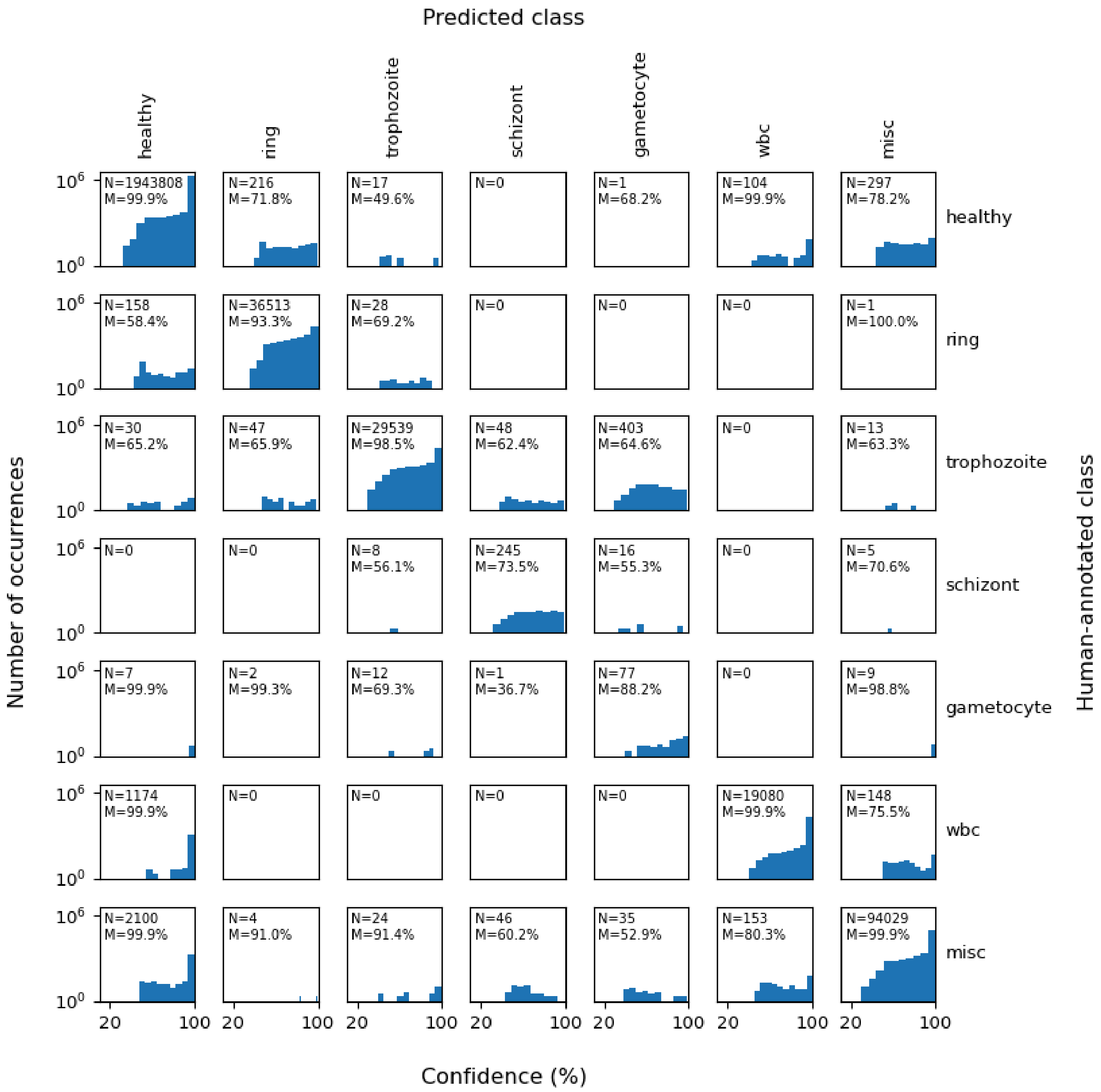

Supplementary Fig. 10 | Matrix of YOGO confidence scores. Each sub-panel is a histogram of model confidence scores for a subset of test data with the given 'Actual class' (human annotated label) and 'Predicted class' (model predicted label), with one-to-one correspondence with the confusion matrix.

medRxiv preprint doi: https://doi.org/10.1101/2024.11.12.24317184; this version posted November 13, 2024. The copyright holder for this preprint (which was not certified by peer review) is the author/funder, who has granted medRxiv a license to display the preprint in perpetuity.
It is made available under a CC-BY 4.0 International license .

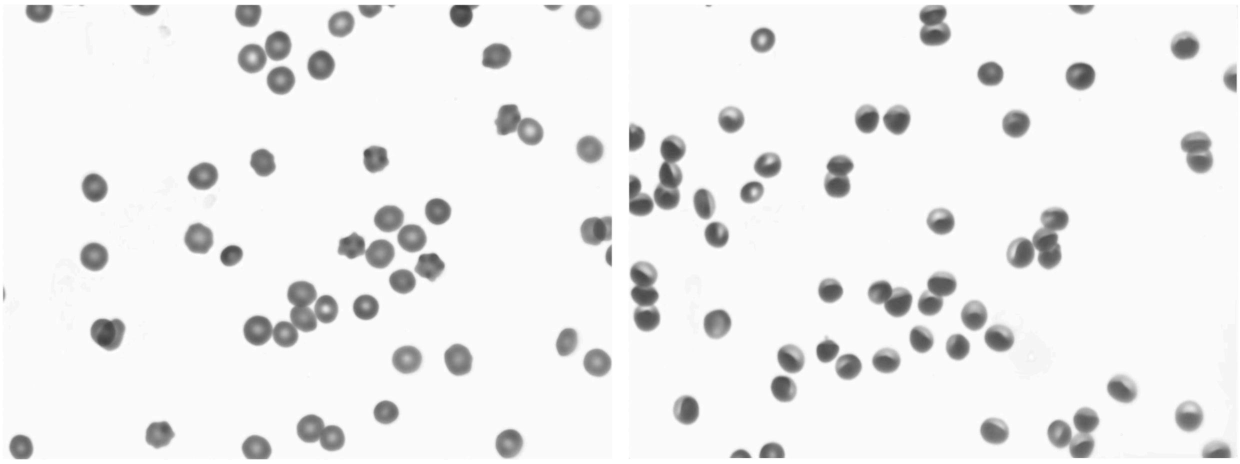

Supplementary Fig. 11 | Effect of flow rate on RBC shape. Diluted blood from a Ugandan cohort participant shown in both stopped (left) and under moderate flow (right). In the stopped condition, RBCs are seen to be primarily discocytes with some type I echinocytes. Under flow, the shear forces cause the cells' leading edges to enrich and their trailing edges to rarify.

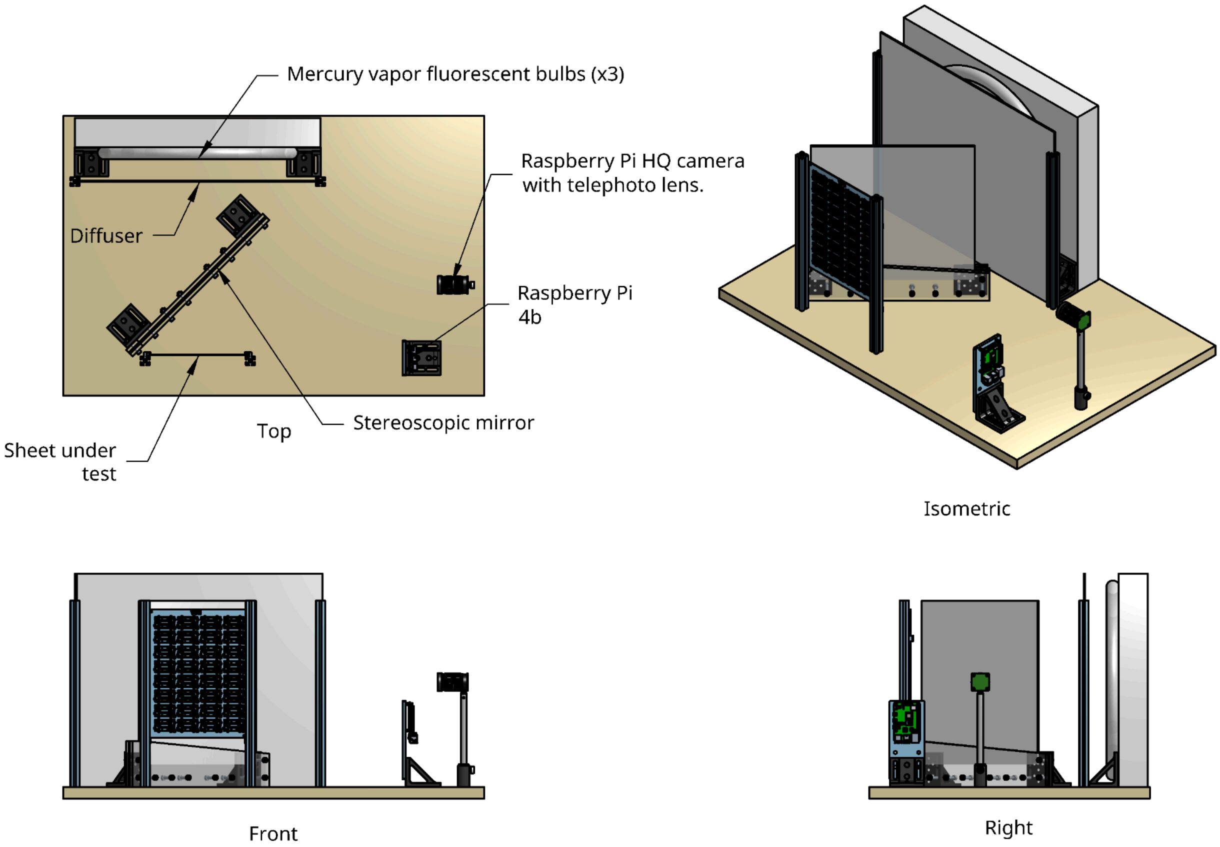

Supplementary Fig. 12 | Design of the flow cartridge quality control interference fringe imager. The imager was constructed by focusing a Raspberry Pi HQ camera with telephoto lens option onto the reflected image of a flow cartridge sheet. The central mirror (purchased from www.stereoscopicmirror.com) was a 14 x 14 inch square, partially-silvered mirror used to transmit fluorescent bulb illumination onto the sheet under test, which was then imaged via reflection onto the camera. The setup permitted uniform illumination of the sheet by three concentric mercury vapor bulbs, which exhibit peaked spectra conducive to forming interference fringes by virtue of the etalon effect within the thin flow cartridge. Interference fringe

medRxiv preprint doi: https://doi.org/10.1101/2024.11.12.24317184; this version posted November 13, 2024. The copyright holder for this preprint (which was not certified by peer review) is the author/funder, who has granted medRxiv a license to display the preprint in perpetuity.
It is made available under a CC-BY 4.0 International license .

patterns were used to assess the flatness of the flow layer, the approximate thickness of the flow layer (presence of strong fringes indicated the chamber was appropriately thin), the wicking of the glue used for bonding, or leaking of glue into the flow channel. Results were assessed in a semi-manual fashion using custom software allowing an operator to score flow cartridges' defects and automatically entering the results into a database.

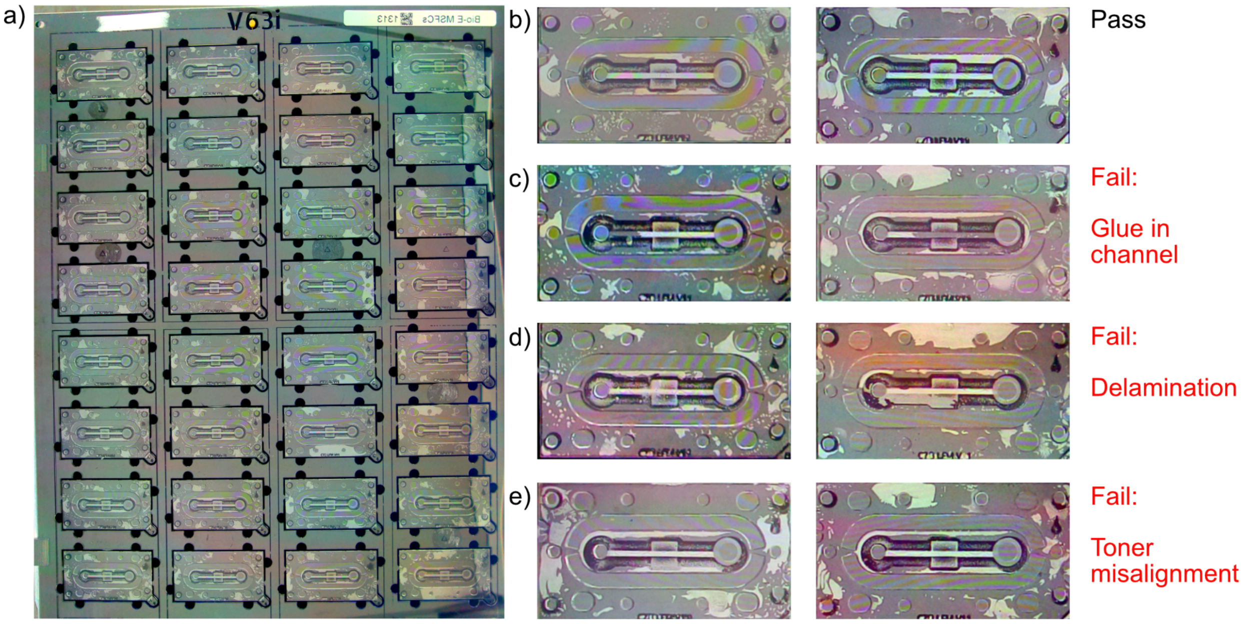

Supplementary Fig. 13 | Example images from the interference fringe imager. a) Whole sheet image from the interference fringe imager. b) Example flow cartridges passing quality control. c) Example flow cartridges failing by presence of glue in the channel. d) Example flow cartridges failing by delamination. e) Example flow cartridges failing by misalignment of the toner pattern.

medRxiv preprint doi: https://doi.org/10.1101/2024.11.12.24317184; this version posted November 13, 2024. The copyright holder for this preprint (which was not certified by peer review) is the author/funder, who has granted medRxiv a license to display the preprint in perpetuity.
It is made available under a CC-BY 4.0 International license .

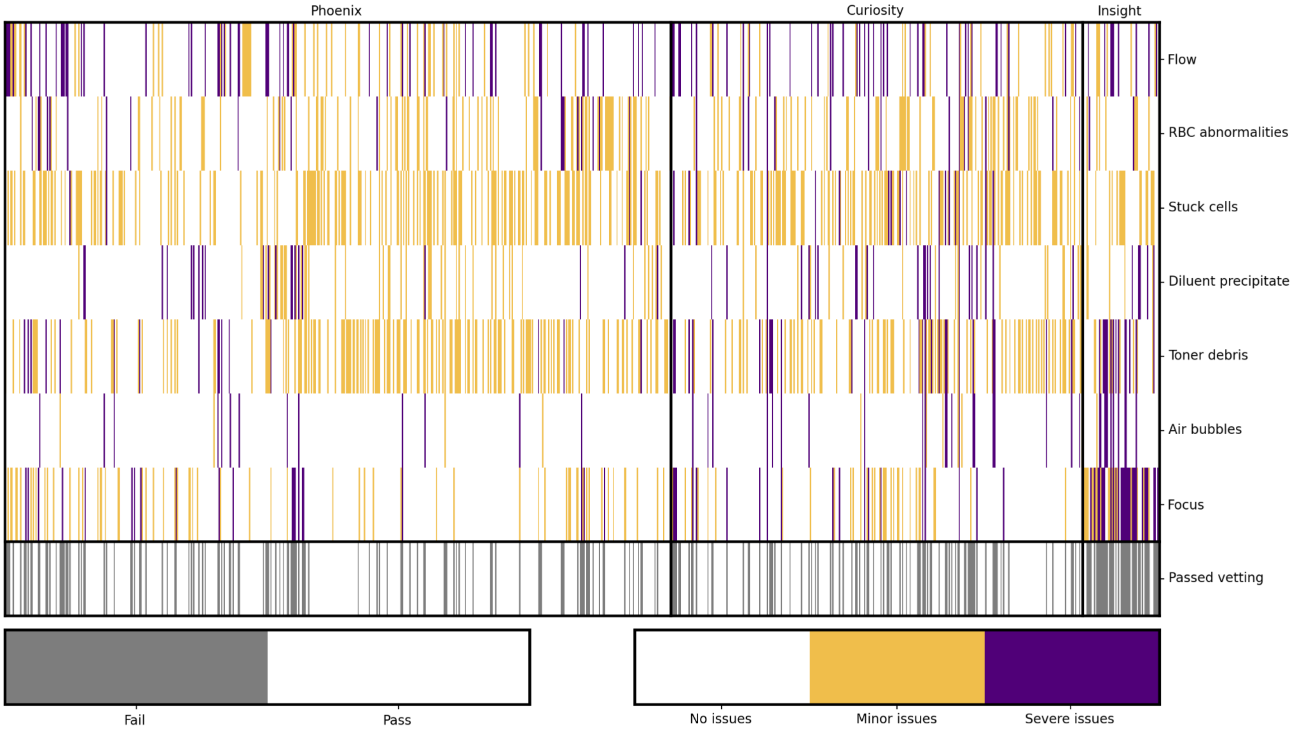

Supplementary Fig. 14 | Manual vetting criteria for quality control of clinical runs. Videos exported from individual experiments in the PRISM border cohort were viewed and scored by the following criteria. Each criteria was subjected to a score of ‘No issues’, ‘Moderate issues’, or ‘Severe issues’. An experiment was rejected if it suffered from one or more severe issues, or if it suffered from four or more moderate issues:

- Flow: Stability of the flow rate.
- Condition of the RBCs: if the majority of RBCs were damaged/lysed the sample was rejected from analysis.
- Stuck cells: if more than ⅓ of the video had ⅓ of the field of view obscured it was considered ‘moderate’. If more than half the field of view was obscured for at least half the video it was considered ‘severe’.
- Diluent precipitate: If reconstituted diluent formed a precipitate that was visible and blocking ⅓ of the field of view it was considered moderate. More than ½ the field of view was considered severe.
- Toner debris: if stray droplets of printer toner (ink) covered the field of view, it was considered either moderate or severe, subject to the reviewer’s discretion.
- Air bubbles: if air bubbles obstructed the field of view, it was considered either moderate or severe, subject to the reviewer’s discretion.
- Focus: Single Shot Auto-Focus (SSAF) functioned the majority of the time, but some videos were degraded by poor focus. Reviewer’s discretion was used to score severity.

medRxiv preprint doi: https://doi.org/10.1101/2024.11.12.24317184; this version posted November 13, 2024. The copyright holder for this preprint (which was not certified by peer review) is the author/funder, who has granted medRxiv a license to display the preprint in perpetuity.
It is made available under a CC-BY 4.0 International license .

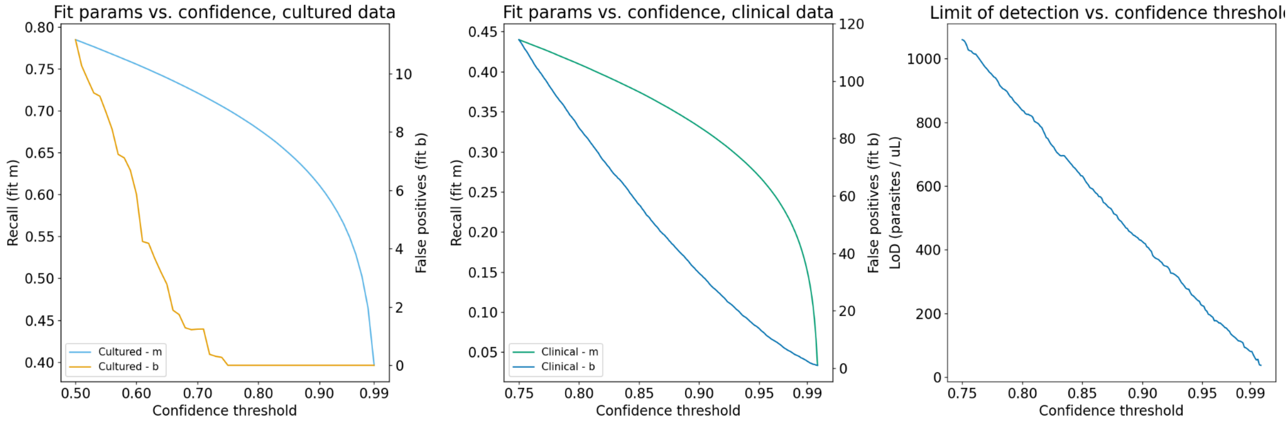

Supplementary Fig 15 | Transformation parameters and limit of detection as a function of model confidence. Linear transformation of the data was performed using fit parameters that were confidence threshold-dependent. With increasing confidence threshold, recall (m) and false positive rate (b) for overall parasitemia both decrease.

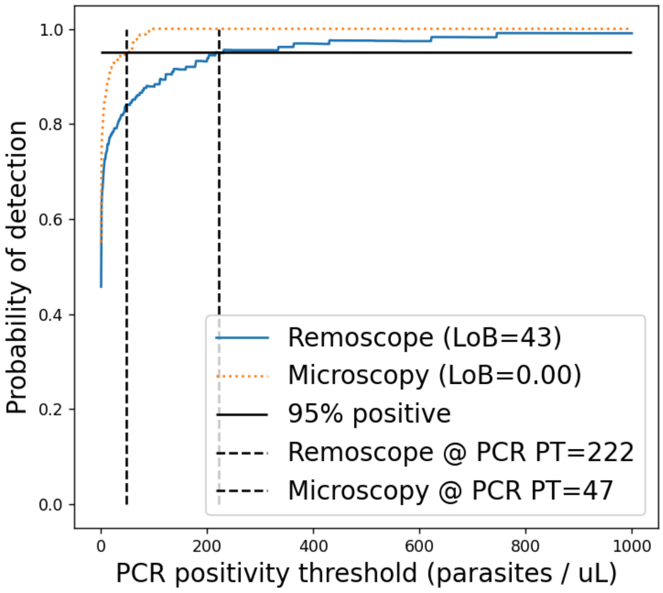

Supplementary Fig. 16 | Probability of detection by Remoscope and microscopy. For each method, the fraction of points scoring above the Limit of Blank (LoB) as a function of parasitemia level. The point at which each method is able to detect 95% of points as positive is highlighted. The LoB for both methods was computed as the mean plus 1.645 standard deviations of all qPCR negative samples.

medRxiv preprint doi: https://doi.org/10.1101/2024.11.12.24317184; this version posted November 13, 2024. The copyright holder for this preprint (which was not certified by peer review) is the author/funder, who has granted medRxiv a license to display the preprint in perpetuity.
It is made available under a CC-BY 4.0 International license .

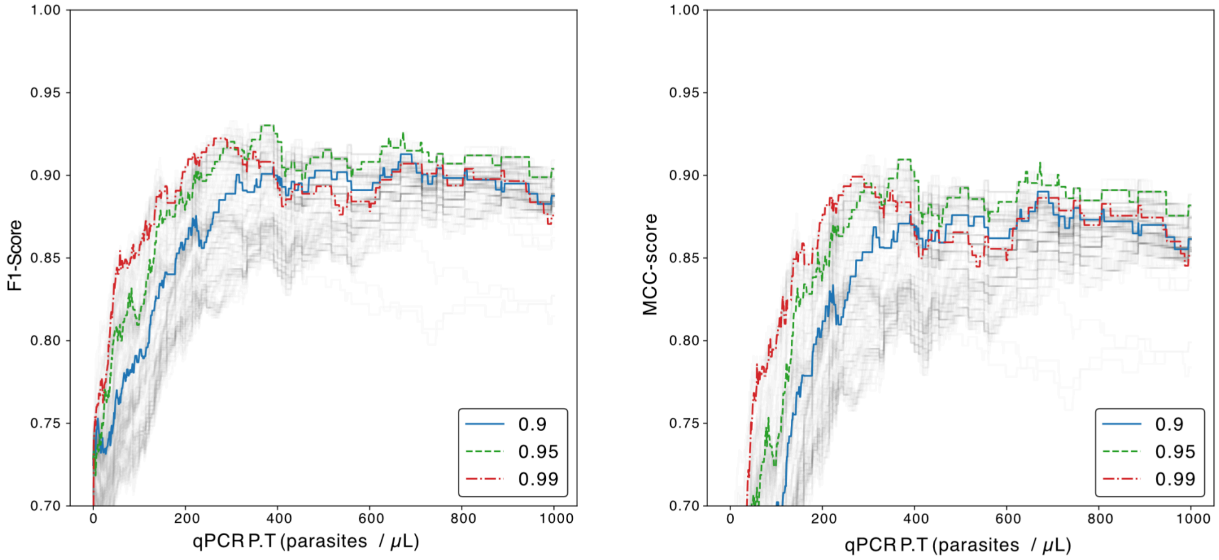

Supplementary Fig. 17 | Remoscope F1 and MCC scores from the clinical cohort diluted blood assay presented as a line chart. Line plots are shown for confidence thresholds between 0.5 and 0.99, with key values highlighted.

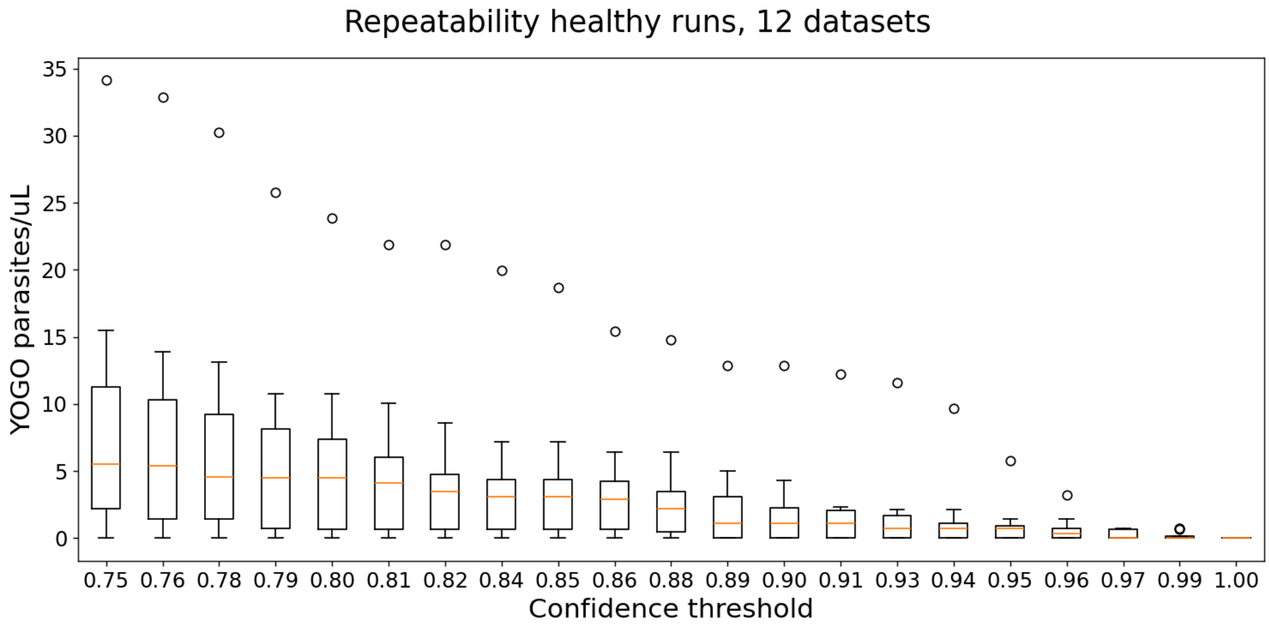

Supplementary Fig 18 | Box plot analysis of false positives as a function of confidence threshold for N=12 experiments from non-endemic donors, using the undiluted blood assay. The box widths represent the interquartile range (IQR), the yellow bands represent the median of the distribution, whiskers extend to the farthest datapoint lying within 1.5 times the IQR, and the individual data markers represent points outside the whiskers.

medRxiv preprint doi: https://doi.org/10.1101/2024.11.12.24317184; this version posted November 13, 2024. The copyright holder for this preprint (which was not certified by peer review) is the author/funder, who has granted medRxiv a license to display the preprint in perpetuity.
It is made available under a CC-BY 4.0 International license .

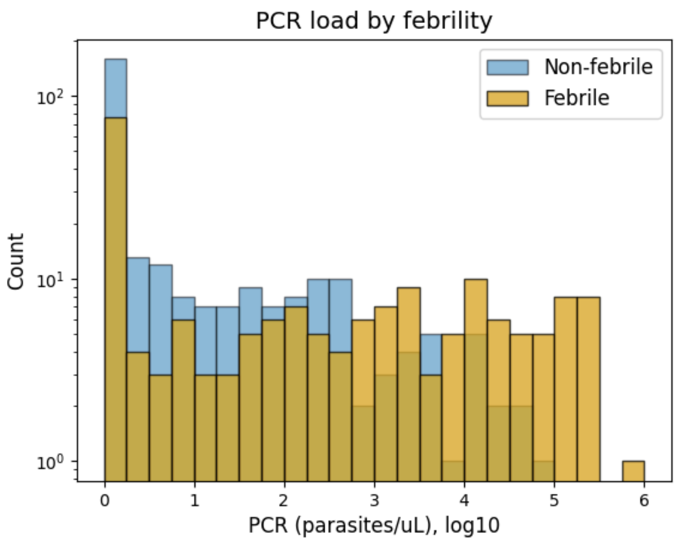

Supplementary Fig. 19 | Histogram of clinical parasitemias by PCR stratified by febrility status. The correlation with febrility increased with high parasitemia levels above (> 1,000 / μL).

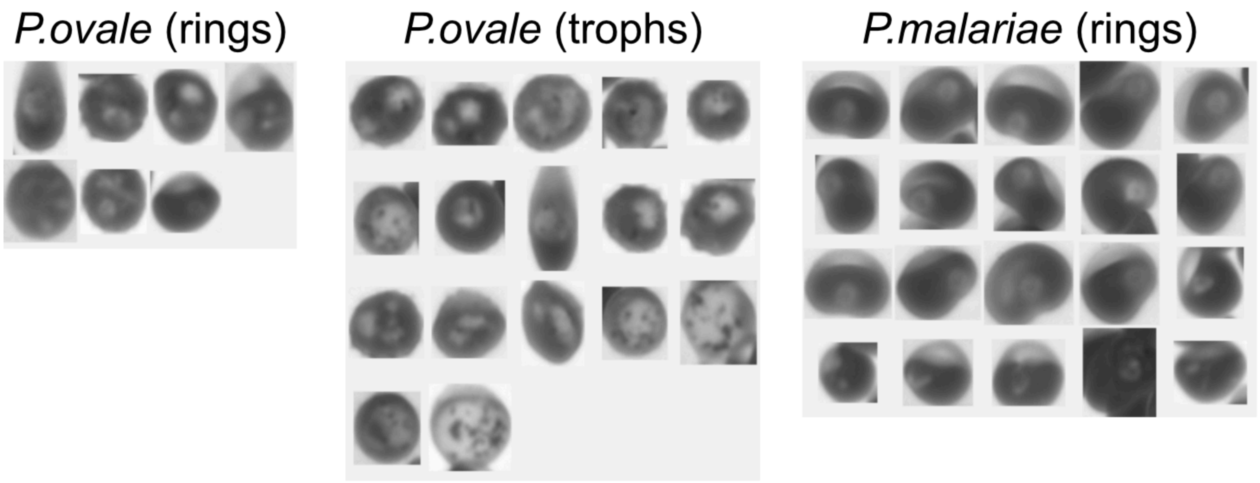

Supplementary Fig. 20 | Putative *Plasmodium ovale* and *Plasmodium malariae*, found by YOGO in the clinical cohort. The indicated species was determined by a non-*falciparum* PCR panel in participants who tested positive by microscopy but negative by *Pf* qPCR reaction. YOGO was not trained to detect these species of parasites.

medRxiv preprint doi: https://doi.org/10.1101/2024.11.12.24317184; this version posted November 13, 2024. The copyright holder for this preprint (which was not certified by peer review) is the author/funder, who has granted medRxiv a license to display the preprint in perpetuity.
It is made available under a CC-BY 4.0 International license .

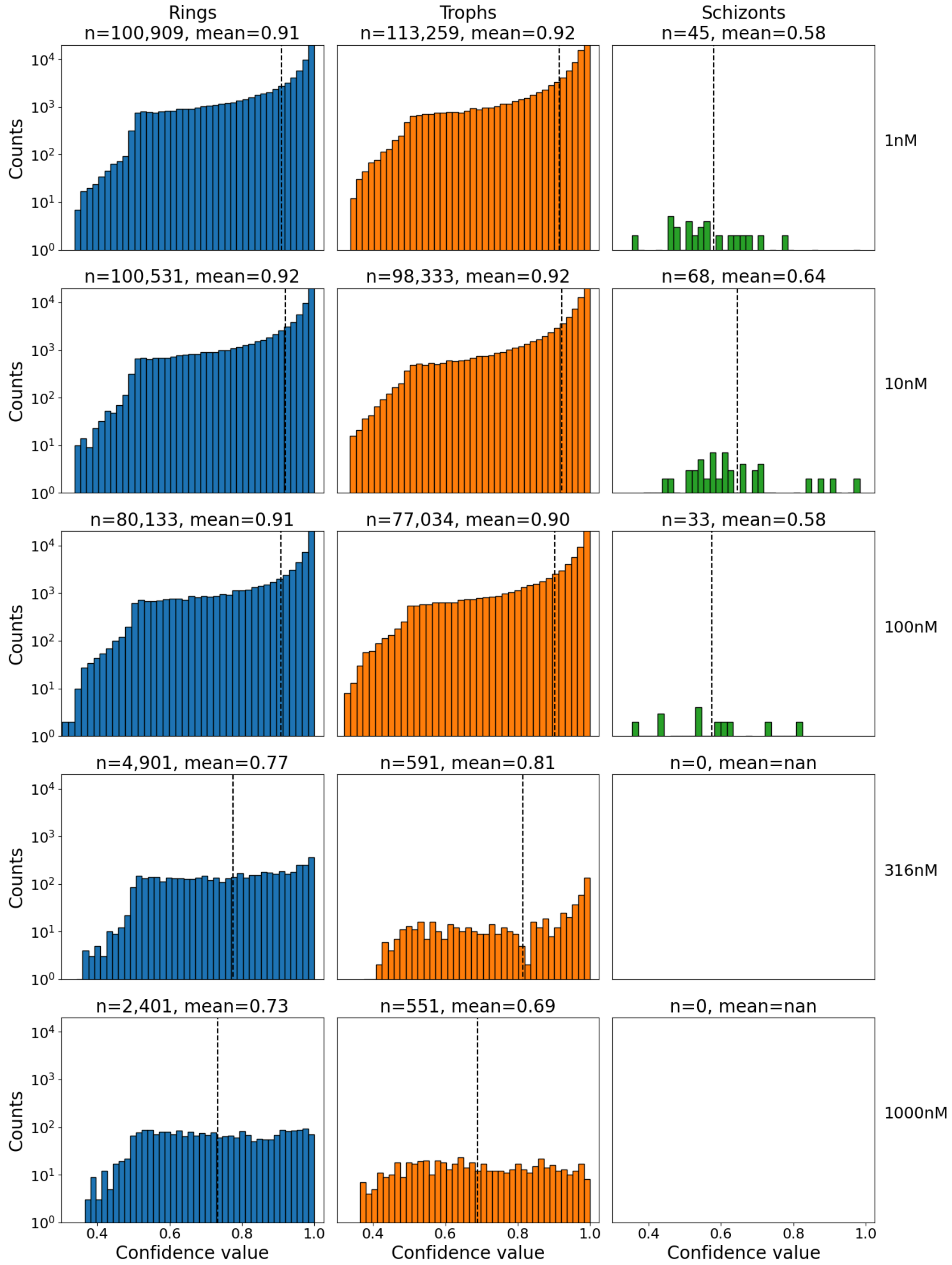

Supplementary Fig. 21 | Model confidence vs drug concentration. Histograms showing YOGO confidence distributions as a function of CQ concentration, for *Pf* rings, trophozoites, and schizonts. YOGO was not trained on drugged parasites, and shows a decrease in mean confidence score in addition to a reduction in total number of detected parasites, consistent with drug-induced parasite morphological changes and death.

medRxiv preprint doi: https://doi.org/10.1101/2024.11.12.24317184; this version posted November 13, 2024. The copyright holder for this preprint (which was not certified by peer review) is the author/funder, who has granted medRxiv a license to display the preprint in perpetuity.
It is made available under a CC-BY 4.0 International license .

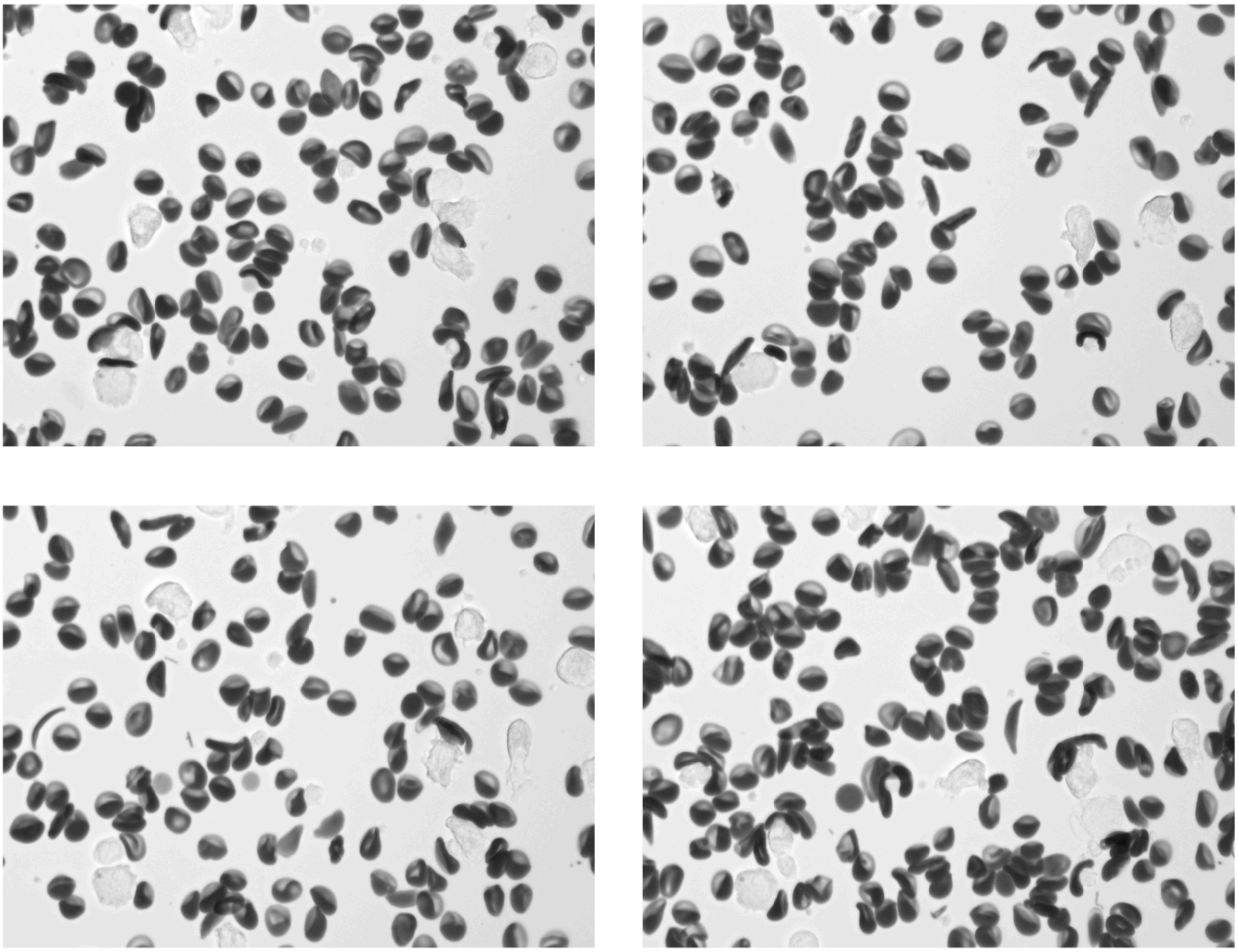

Supplementary Fig. 22 | Example images from a participant with Sickle Cell Disease (SCD). While some cells exhibit well-known sickling behavior, the majority of RBCs exhibit a diversity of abnormal morphologies.

medRxiv preprint doi: https://doi.org/10.1101/2024.11.12.24317184; this version posted November 13, 2024. The copyright holder for this preprint (which was not certified by peer review) is the author/funder, who has granted medRxiv a license to display the preprint in perpetuity.
It is made available under a CC-BY 4.0 International license .

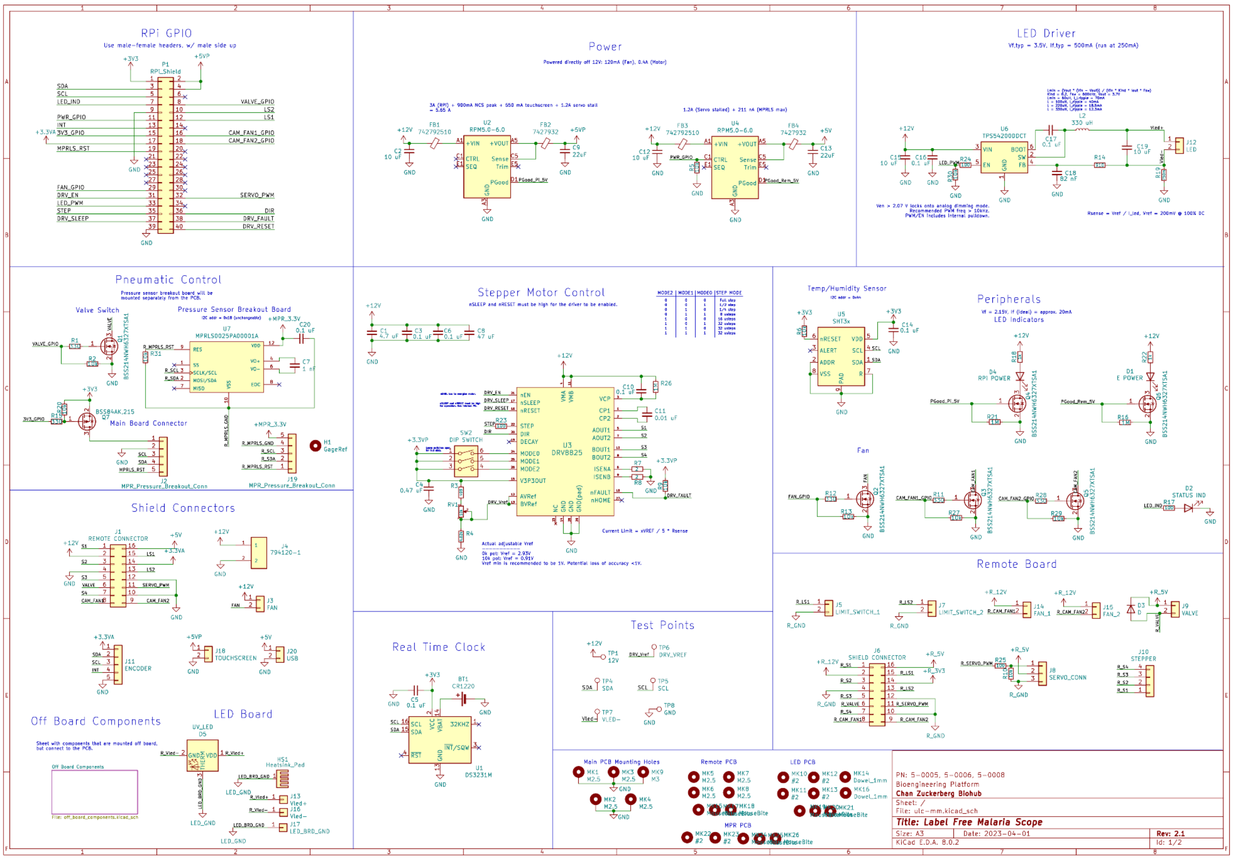

Supplementary Fig. 23 | Remoscope main and daughter board PCB design schematic. This diagram contains all components present on the main board, daughter board, LED board, and pressure sensor board. See https://github.com/czbiohub-sf/remo-pcbs for full design details including PCB layout.

medRxiv preprint doi: https://doi.org/10.1101/2024.11.12.24317184; this version posted November 13, 2024. The copyright holder for this preprint (which was not certified by peer review) is the author/funder, who has granted medRxiv a license to display the preprint in perpetuity.
It is made available under a CC-BY 4.0 International license .

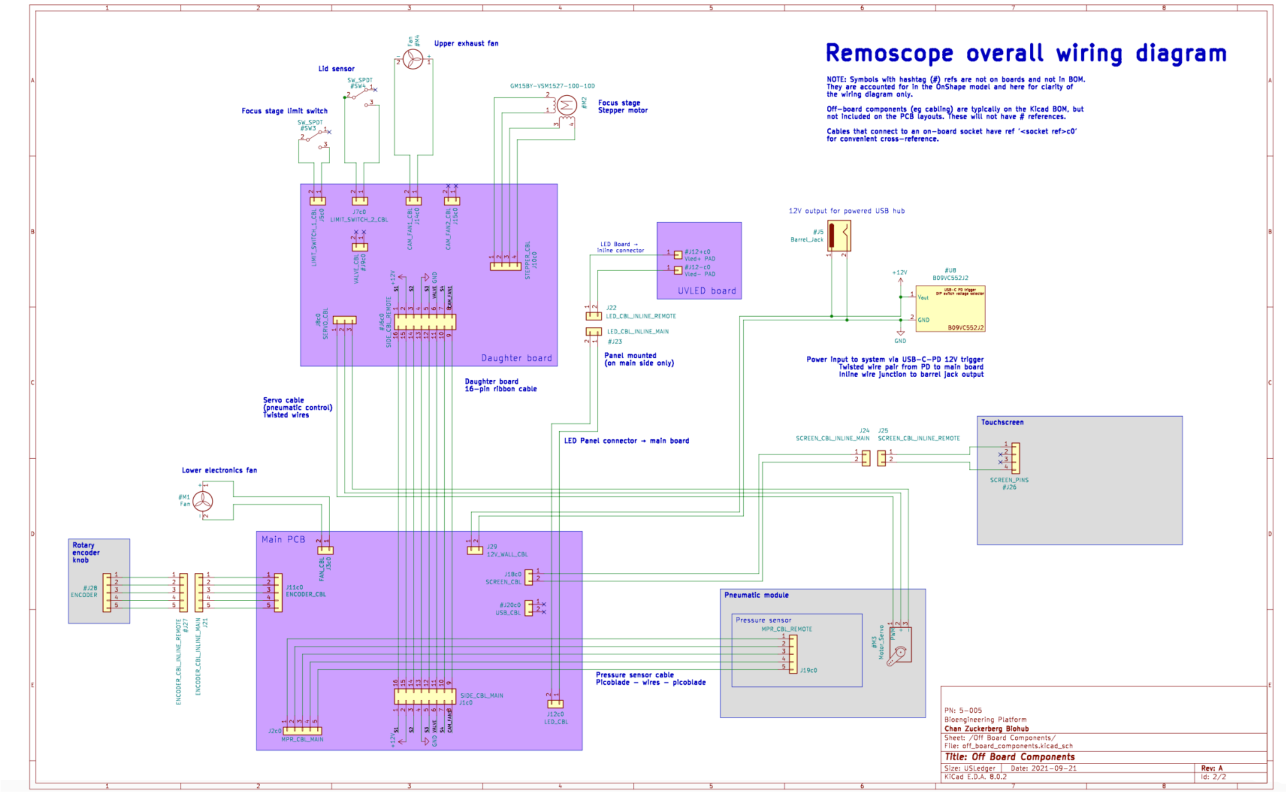

Supplementary Fig. 24 | Remoscope overall wiring diagram. Shaded purple boxes represent custom printed circuit boards, and shaded beige boxes represent other modules within the system, and groups of wires represent custom cabling solutions.
